# Physiological responses, yield and medicinal substance (andrographolide, AP1) accumulation of *Andrographis paniculata* (Burm. f) in response to plant density under controlled environmental conditions

**DOI:** 10.1371/journal.pone.0272520

**Published:** 2022-08-04

**Authors:** Panita Chutimanukul, Kriengkrai Mosaleeyanon, Supattana Janta, Theerayut Toojinda, Clive Terence Darwell, Praderm Wanichananan

**Affiliations:** National Center for Genetic Engineering and Biotechnology (BIOTEC), National Science and Technology Development Agency, Pathum Thani, Thailand; National University of Kaohsiung, TAIWAN

## Abstract

Agricultural practice in adjusting planting density and harvest date are important factors for plant development and crop improvement, reaching maximum yields and enhancing the production of secondary metabolites. However, it is unclear as to the optimal planting densities during mass production that encourage consistent, high yield secondary metabolite content. For this, controlled environment, crop production facilities such as plant factories with artificial lighting (PFAL) offer opportunity to enhance quality and stabilize production of herbal plants. This study assessed the effect of plant density and harvest date on physiological responses, yield and andrographolide (AP1) content in *Andrographis paniculata* (Andrographis) using hydroponic conditions in a PFAL system. Andrographis, harvested at vegetative stage (30 days after transplanting; 30 DAT) and initial stage of flowering (60 DAT) exhibited no significant differences in growth parameters or andrographolide accumulation according to planting densities. Harvest time at flowering stage (90 DAT) showed the highest photosynthetic rates at a planting density of 15 plants m^-2^. Highest yield, number of leaves, and Andrographolide (AP1) content (mg per gram of DW in m^2^) were achieved at a more moderate planting density (30 plants m^-2^). Finally, five out of seventeen indices of leaf reflectance reveal high correlation (*r* = 0.8 to 1.0 and *r* = -0.8 to -1.0, P<0.01) with AP1 content. These results suggest that a planting density of 30 plants m^-2^ and harvest time of 90 DAT provide optimal growing condition under the hydroponic PFAL system.

## Introduction

*Andrographis paniculata* (Acanthaceae; Burm. F.) Wall. ex Nees, commonly known as "King of Bitters”, is an annual herbaceous plant distributed throughout tropical and subtropical Asia, Southeast Asia, and India [1]. *A*. *paniculata* contains diterpenoids, flavonoids and polyphenols as major bioactive compounds [2, 3]. Andrographolide (C_20_H_30_O_5_; AP1) is the main diterpenoid in *A*. *paniculata*, making up about 4%, 0.8–1.2% and 0.5–6% (w/w) in dried whole plants, stems, and leaf extracts, respectively [4–6]. The other main diterpenoids are deoxyandrographolide, neoandrographolide, 14-deoxy-11,12-didehydroandrographolide and isoandrographolide [5, 7]. Andrographolide is a potential chemotherapeutic agent showing various pharmacological properties. It has extremely bitter taste, is colorless and crystalline in appearance, and possess a “lactone function” [8]. AP1 reportedly shows cytotoxic activity against human cancer cells (KB: human epidermoid carcinoma and P388: lymphocytic leukaemia) [9]. Moreover, research during the last few decades has indicated that Andrographis extract is useful as an antiviral [10], anti-myocardial infraction [11] and anti-inflammatory [12].

Secondary metabolites, despite their genetic basis, are strongly affected by environmental influences [13]. Agricultural factors, such as spacing and harvesting time, have a critical effect on quantitative and qualitative characteristics determining plant growth and yield increment. Previous studies have shown that intraspecific interactions can affect characteristics of plant canopy [14], roots morphology [15] and plant metabolic levels [16], all ultimately affecting plant growth and quality [17]. The effects of intraspecific interactions on plant performance can be easily realized by altering planting density [18, 19]. Thus, obtaining high crop productivity depends on improved understanding of crop requirements. Planting density significantly affects plant growth, development and biomass [20, 21], while high planting densities result in greater stresses on plant communities and reduced resource availability which may reduce accumulation of secondary metabolites [22, 23]. Shalby and Razin [23] showed that planting density influences growth, biomass and essential oil content in thyme (*Thymus* spp). Moreover, plant density influences leaf area, which impacts light interception and canopy photosynthesis [24]. In contrast, narrow row spacing produced a larger yield than wider rows due to the better interception of light in soybean cultivation [25, 26]. Zhang [27] also reported that cotton canopy photosynthetic rates increased with plant density, at a cost of reduced leaf area and yield.

To understand these dynamics, plant response and biochemical parameters according to agricultural factors, such as plant biomass, pigment content, phenolic content, can be estimated by leaf reflectance from visible light spectra the plant status from visible (400–700 nm) and near infrared (700–1,000 nm) [28, 29]. In order to assess relationships between spectral reflectance and plant response characteristics, vegetation indices that correlate plant growth and plant responses by evaluating measurements from multiple spectral bands have been devised [30]. One of the most commonly used is normalized difference vegetation indices (NDVI), that incorporates growth traits and general plant condition (i.e., green biomass, plant health and nitrogen content on the plant canopy) [31, 32].

For use in the pharmaceutical industry, plant Andrographolide content tends to be inconsistent due to variation in environmental conditions at different locations and genetic variation among plant stock. Both growing region and seasonal change have a strong impact on formation of diterpene lactones. Plant factories with artificial lighting (PFAL) are among the most advanced of modern agricultural technologies that mitigates production inconsistency by systematically controlling sowing, cultivating, and harvesting, within a regulated indoor space [33, 34]. Growing crops in rigorously controlled conditions presents significant advantages over traditional farming because it guarantees more predictable year-round yields and cost savings due to automated practices. It is recognized that plant space and harvest time are critical variables for PFAL optimization.

There has been little research on the development of agrotechniques for industrial agriculture. A standard planting distance and harvest period for this crop is desirable. Not only will optimizing planting density and harvesting time enhance yield and quality, but it will also reduce input costs by decreasing seed rate and fertilizer consumption [35]. The goal of this study was to investigate the hypothesis that planting density will affect physiological responses, growth, yield and AP1 contents in *A*. *paniculata* grown in a hydroponic system under PFAL, and the conceptual framework of the study is shown in S1 Fig. Such knowledge will contribute to the advancement of precision farming practices.

## Materials and methods

### Plant material and growth conditions

**Fig 1:**
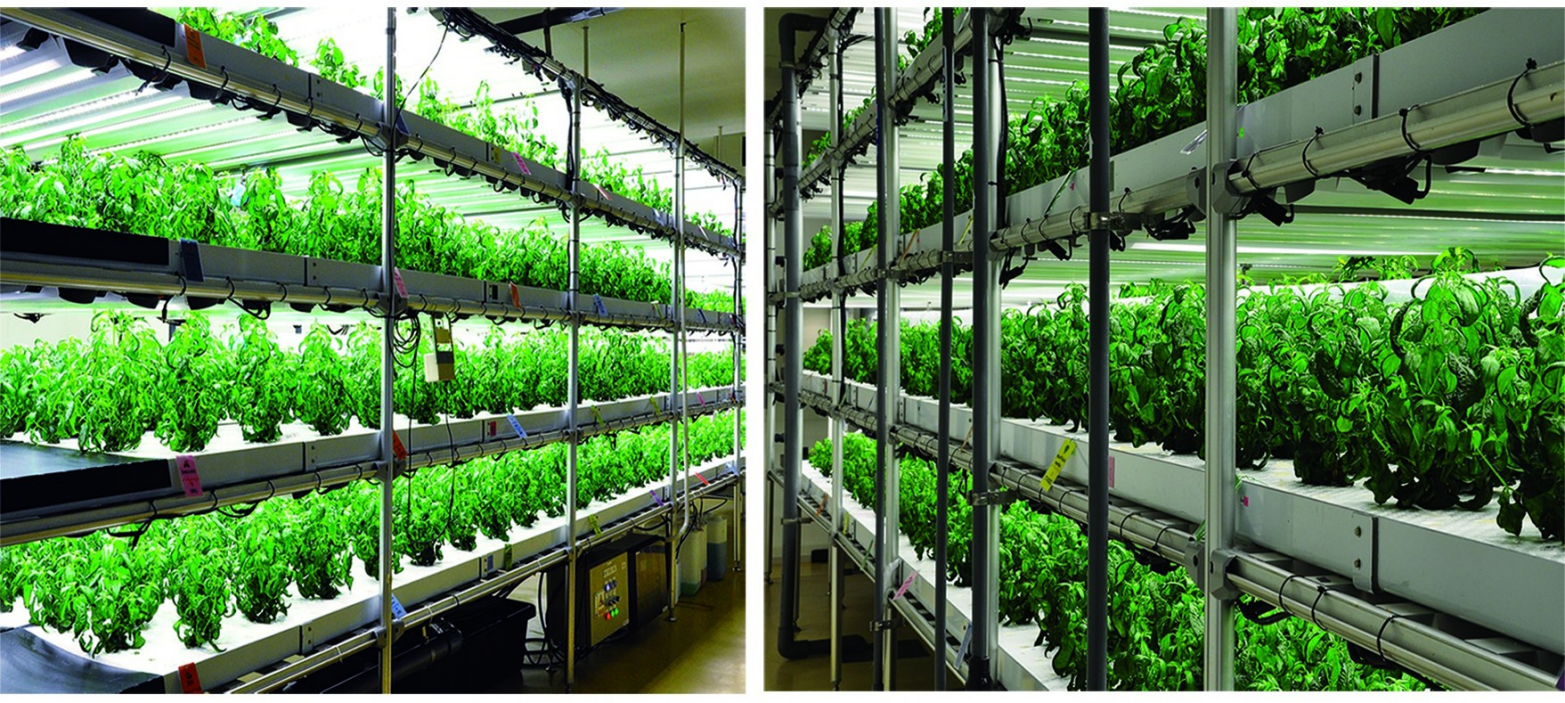
Andrographis plants grown in hydroponic PFAL system with artificial lighting.

The experiment was conducted in the plant factory (PFAL) facility at the National Center for Genetic Engineering and Biotechnology (BIOTEC), National Science and Technology Development Agency (NSTDA), Thailand. Seeds of Andrographis (*Andrographis paniculata*) were purchased from a commercial seed company (Benjamitr Enterprise (1991) Co. Ltd., Nonthaburi, Thailand). Seeds were sown in a 96-cell germination sponge (23×32×3 cm) and germinated in transplantation room using the following conditions: a photosynthetic photon flux density (PPFD) of 100 μmol m^-2^ s^-1^ of white LED lights under a 16 h d^-1^ photoperiod (AGRI-OPTECH Co., Ltd, Taiwan), 380 ± 50 μmol mol^-1^ CO_2_, air temperature of 25 ±1°C, with 75 ± 5% of relative humidity (RH). On day 30 after sowing, all seedlings with well-developed roots and first true leaf pair were transplanted into a deep-flow-technique hydroponic system in the PFAL. Andrographis plants were cultivates in a cultivation room comprising towers (120 cm width x 540 cm height x 900 cm length) each with four shelves (each cultivation shelf is 45 cm tall) (Fig 1). Plant growth conditions were set as follows: 200 μmol m^-2^ s^-1^ of white LEDs, with 16 h d^-1^ photoperiod, 25 ± 1°C air temperature, 75 ± 5% RH, and 1,000 ± 100 μmol mol^-1^ CO_2_ (for details of the daily environment under PFAL, see S1 Table). Seedlings were irrigated with modified Enshi nutrient solution [36] (1:200) consisting of 190 g L^-1^ Ca(NO_3_)_2_, 162 g L^-1^ KNO_3_, 98 g L^-1^ MgSO_4_, 30.8 g L^-1^ NH_4_H_2_PO_4_, 4 gL^-1^ Fe-EDTA, 5 g L^-1^ micronutrient, 0.572 g L^-1^ H_3_BO_3_, 0.422 g L^-1^ MnSO_4_·4H_2_O, 0.044 g L^-1^ ZnSO_4_·7H_2_O, 0.016 g L^-1^ CuSO_4_·5H_2_O, 0.005 g L^-1^ NaMoO_4_·H_2_O. The EC and pH of the nutrient solution were adjusted automatically and maintained at 1.5 dS m^-1^ and 5.7, respectively.

### Physiological and growth measurements

**Fig 2:**
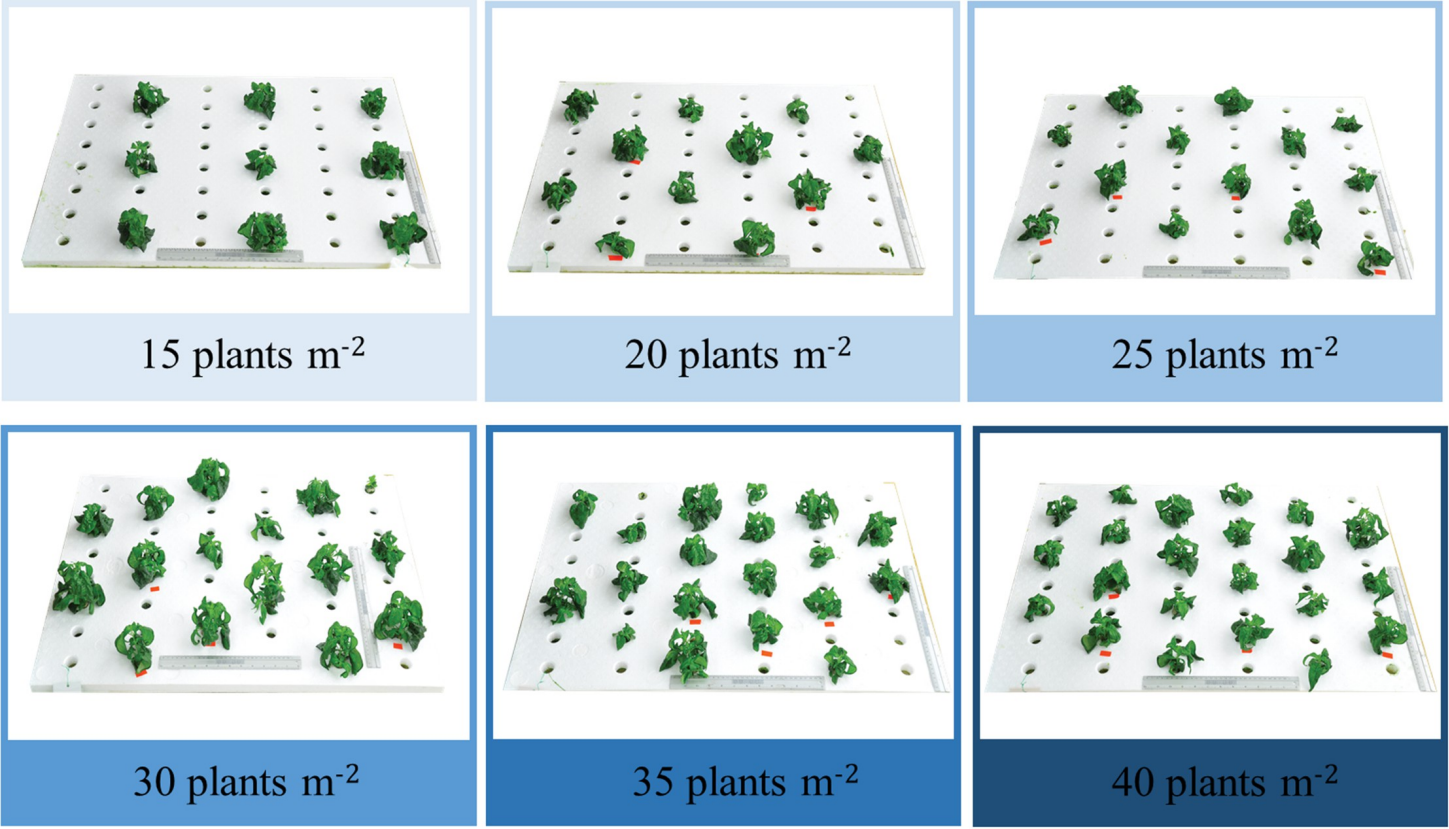
Experimental design of Andrographis at six planting densities in a PFAL. Plant cultivation on a floating foam (60×90 cm) includes the following densities: 15, 20, 25, 30, 35 and 40 plants m^-2^.

To evaluate the physiological responses of different planting densities (15, 20, 25, 30, 35 and 40 plants m^-2^; Fig 2), the following characteristics were estimated at 30 (vegetative stage), 60 (initial stage of flowering) and 90 (flowering stage) days after transplantation (DAT). Photosynthetic parameters of Andrographis plants were measured at each planting density using 4 replicates (three plants per replication) with a portable photosynthesis system (LI-6800, LICOR Inc., Lincoln, NE). Photosynthetic rate (*Pn*), stomatal conductance (*gs*), internal concentration (*Ci*), and transpiration (*E*) were measured on fully expanded leaves located at the fourth node from the top using the following conditions: CO_2_ concentration: 1,000 μmol mol^-1^, the molar flow of air per unit leaf area: 500 mmol m^-2^ s^-1^, leaf temperature: 25°C, relative humidity: 75%, and leaf surface photosynthetically active radiation: 300 μmol m^-2^ s^-1^. A light spectrum ratio of red and blue at 1:1 was applied. The reflectance spectra of Andrographis leaves were measured with a PolyPen RP 400 UVIS (Photon Systems Instruments, Prague, Czech Republic). For measuring reflectance spectra, four Andrographis leaves on the second, third, fourth and fifth node from the top of the plant were placed individually into a clip of the PolyPen measuring head. Seventeen reflectance indices (Table 1) were calculated, and plant height and canopy width were measured with a ruler. The numbers of leaves and stalks were counted manually. After harvesting, both fresh weight (FW) and dry weight (DW) of above-ground yields were determined. The yield of mass was calculated based on number of whole plants per m^2^.

**Table 1:** Vegetation indices used for Andrographis leaf characteristic assessment.

Index	Description	Formulation	Reference
NDVI	Normalized Difference Vegetation Index	(NIR–RED) / (NIR + RED)	[37]
MCARI1	Modified Chlorophyll Absorption in Reflectance Index	1.2 * [2.5 * (R790 –R670)– 1.3 * (R790 –R550)]	[38]
OSAVI	Optimized Soil-Adjusted Vegetation Index	(1 + 0.16) * (R790 –R670) / (R790 –R670 + 0.16)	[39]
G	Greenness Index	R554 / R677	[40]
MCARI	Modified Chlorophyll Absorption Ratio *Index*	[(R700 –R670)– 0.2 * (R700 –R550)] * (R700 / R670)	[41]
TCARI	Transformed CAR Index	3 * [(R700—R670)– 0.2 * (R700-R550) * (R700 / R670)]	[42]
TVI	Triangular Vegetation Index	0.5 * [120 * (R750 –R550)– 200 * (R670 –R550)]	[43]
SPRI	Simple Ratio Pigment Index	R430 / R680	[44]
NPQI	Normalized Phaeophytinization Index	(R415 –R435) / (R415 + R435)	[45]
PRI	Photochemical Reflectance Index	(R531 –R570) / (R531 + R570)	[46]
NPCI	Normalized Pigment Chlorophyll Index	(R680 –R430) / (R680 + R430)	[44]
Ctr2	Carter Index	R695 / R760	[47]
Lic2	Lichtenthaler Index	R440 / R690	[48]
SIPI	Structure Intensive Pigment Index	(R790 –R450) / (R790 + R650)	[44]
GM2	Gitelson and Merzlyak Indexe	R750 / R700	[49]
ARI2	Anthocyanin Reflectance Index	R800 * (1 / R550–1 / R700)	[50]
CRI2	Carotenoid Reflectance Index	(1 / R510)–(1 / R700)	[51]

### Estimation of andrographolide (AP1) content

#### Sample extraction

After harvesting at each timepoint, whole plants (leaf, stem, and inflorescence) were freeze dried under SP VirTis Genesis Pilot Lyophilizer (SP Scientific, U.S.A.) for 48 hours. Plant extractions were performed on dried tissue using a modified extraction protocol [52]. Each dried sample was ground into a fine powder using a mortar and pestle until forming a fine powder. Plant extraction was conducted on 120 mg of fine powder with 10 mL of methanol (Methanol, 99.9%, HPLC, FISHER). The extracted solution was absolutely mixed and sonicated by ultrasonic cleaner (Bransonic, Branson, Germany) for 30 minutes. Then, the mixture was centrifuged at 5,000–7,000 rpm (Benchtop centrifuge 5810 R, Eppendorf, U.S.A.) for 5 min, filtered through filter paper No.1 (Whatman) and evaporated in methanol at 40°C with evaporator using a Genevac Rocket Centrifugal Evaporator (SP Scientific, U.S.A.). The crude extract was dissolved in 5 mL of 5% methanol (Methanol, HPLC, FISHER) then purified through a C18 solid-phase extraction Florisil 6 cc column (Waters, U.S.A.). The supernatant was diluted in 5 mL of 80% methanol at the volume ratio of 1:10. Prior to analysis, all extract samples were filtered with 0.22 μm syringe filters and stored at -20°C for subsequent analysis.

#### Andrographolide quantification

In order to investigate plant density effects on AP1 accumulation of Andrographis under controlled environment. AP1 content in each sample was analyzed by high performance liquid chromatography (HPLC) (UltiMate 3000 UHPLC system, Thermo Scientific, U.S.A) combined with a photodiode array detector (Dionex UltiMate 3000 Diode Array Detector, Thermo Scientific, U.S.A.) and equipped with ODS Hypersil C18 column Dia. 250×4.6 mm, Particle Size 5 μm (Hypersil GOLD C18 HPLC Columns, Thermo Scientific, U.S.A.) using acetonitrile (Acetonitrile HPLC, FISHER) and deionized water at a flow rate 1.0 mL min^-1^. The injection volume of the sample was 10 μL and detection was performed through a UV probe at a wavelength of 206 nm for 30 minutes. AP1 content was calibrated by comparison with andrographolide standard solution (Sigma-Aldrich). The yield of andrographolide content was shown as milligrams per gram of dry weight and calculated based on number of whole plants per m^2^.

### Experimental design and statistical analyses

All experiments were conducted using four replicates, each consisting of three plants with completely randomize design (CRD). Statistical analysis was analyzed with SPSS (IBM Corporation; Armonk, NY, USA). One-way analysis of variance (ANOVA) was used to examine the differences between treatments for each parameter. Statistical differences between treatments were analyzed with Duncan’s multiple range test (DMRT) tested at the p < 0.05 level. The data presented are the mean ± SE (standard error) of four replicates for each group.

In order to investigate the association between different variables at six different planting densities and three harvest times, a principal component analysis (PCA) was performed using the software JMP version 6.0 (SAS Institute Inc., Cary, NC, USA). In addition, Pearson’s correlation coefficient was used to evaluate the relationship between physiological response, growth, yield and AP1 content. Further, a hierarchical cluster analysis was performed using Ward’s method which provides output results in heat map format. For this, Z-scores were calculated by subtracting the actual value from the mean score of each parameter and dividing by the standard deviation of its parameters.

## Results

### Photosynthetic gas exchange performance

**Fig 3:**
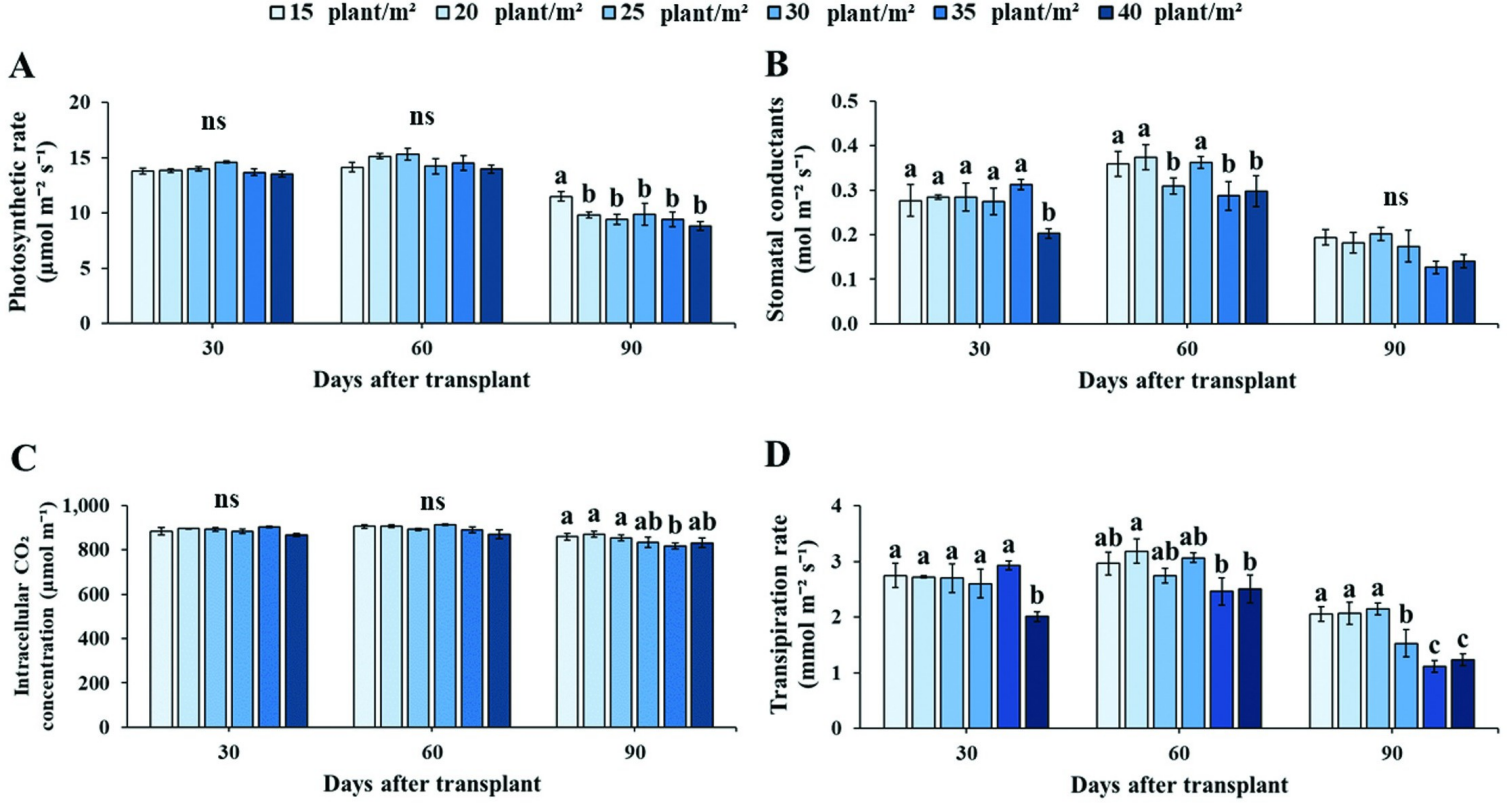
Responses of gas exchange parameters: net photosynthesis rate (*Pn*) (A), stomatal conductance (*gs*) (B), internal CO_2_ concentration (*Ci*) (C) and transpiration rate (*E*) (D) of Andrographis leaves at six planting densities during vegetative (30 DAT), initial flowering (60 DAT) and flowering (90 DAT) stages. Values are represented as mean ± SE (*n* = 4). Different letters indicate significant differences between planting densities at p < 0.05. “ns” indicates no significant difference.

Plant density conditions influenced several photosynthetic gas exchange parameters of Andrographis plants grown under PFAL (Fig 3). Net photosynthesis rate (*Pn*) of Andrographis leaves at various planting densities was not significantly different among the plant densities at vegetative and initial stage of flowering (Fig 3A). In contrast, *Pn* of Andrographis leaves at flowering stage were significantly different according to plant densities, with 15 plant m^-2^ recording the highest *Pn* values. The higher planting densities (20, 25, 35 and 40 plants m^-2^) caused a significant decrease in the *Pn* value of Andrographis leaves. Moreover, results show that *Pn* values were higher in vegetative and initial stage of flowering compared to flowering stage.

During the developmental stage of plant growth, Andrographis leaves was significantly different in stomatal conductance (*gs*) under six planting densities (Fig 3B). The highest reduction in *gs* was found at the highest planting density (40 plants m^-2^) at vegetative stage. At initial stage of flowering, *gs* was significantly higher at 15, 20 and 30 plants m^-2^ than at the moderate density of 25 plants m^-2^ and at high densities (35 and 40 plants m^-2^). However, *gs* values of Andrographis at flowering stage were not significantly different across planting densities (Fig 3B). Conversely, internal CO_2_ concentration (*Ci*)levels of Andrographis during vegetative and early flowering stages were not significantly different, while plants at 35 plants m^-2^ showed the lowest values of *Ci* when compared with other densities during initial stage of flowering (Fig 3C). Transpiration rate (*E*) of Andrographis plants was significantly influenced by planting density (Fig 3D). A decrease in *E* was identified among developmental stages under high densities, especially at 35 and 40 plant m^-2^, which showed the highest reduction of *E*. Moreover, *E* of Andrographis leaves showed similar trends as for *gs* values during all three developmental stages and showed the highest values under 15, 20 and 25 plants m^-2^ (Fig 3D).

### Plant growth, productivity and andrographolide (AP1) accumulation

**Fig 4:**
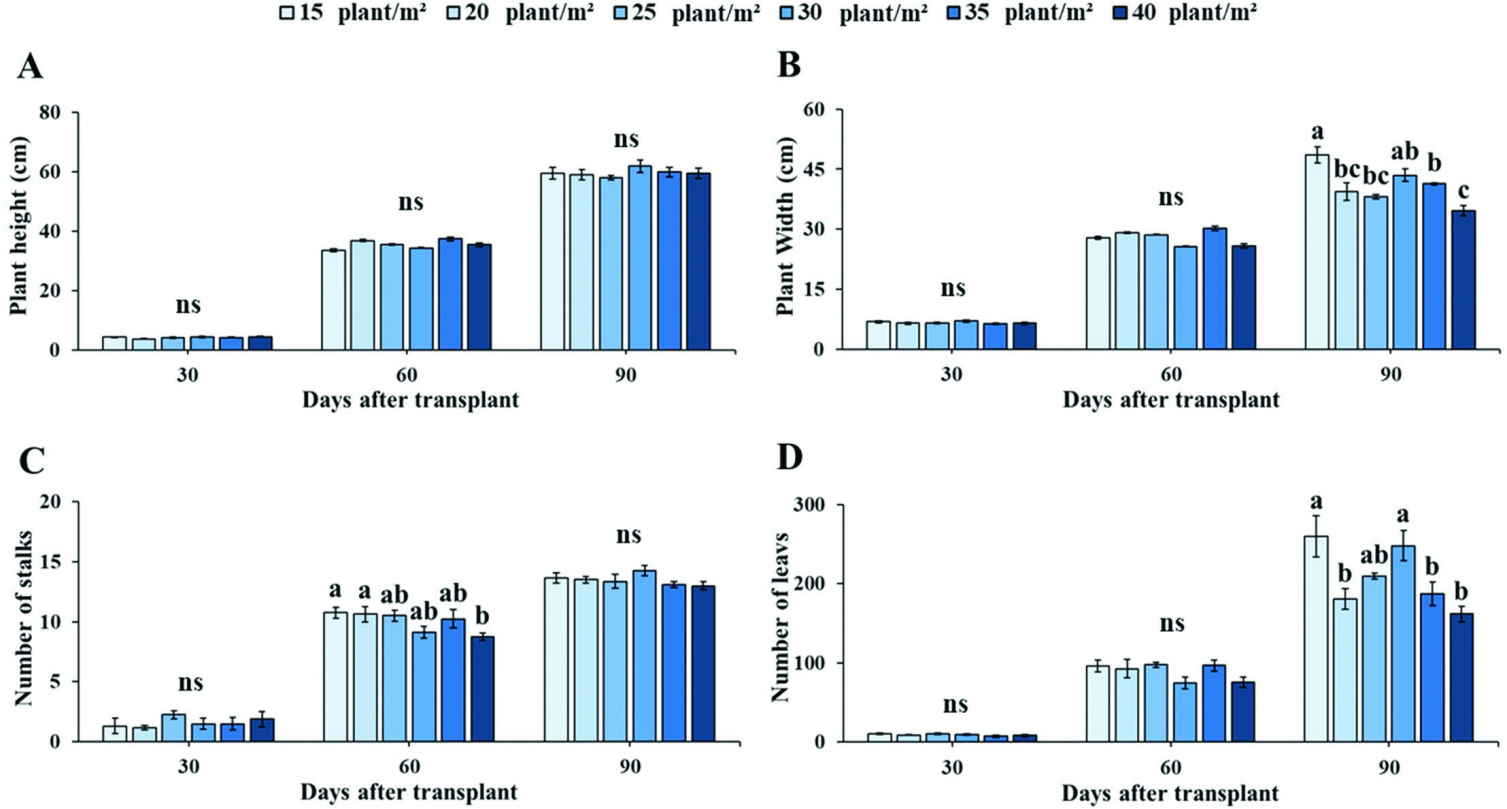
Effects of planting densities on plant growth. Plant height (A), plant width (B), number of stalks (C) and number of leaves (D) of Andrographis during vegetative (30 DAT), initial flowering (60 DAT) and flowering (90 DAT) stages. Values are represented as mean ± SE (*n* = 4). Different letters indicate significant difference between planting densities at p < 0.05. “ns” indicates no significant difference.

In order to investigate plant density effects on agronomic characters and yield, plant height, plant width, number of stalks, number of leaves, fresh and dried weight were examined. Plant density treatments did not significantly affect plant height at different development stages (Fig 4A). At flowering stage, plant width decreased with increased planting density. The largest plant width was recorded at 15 plants m^-2^ (48.56 ± 3.32 cm), which was significantly greater than of 20, 25, 35 and 40 plants m^-2^. The lowest width (34.56 ± 1.74 cm) was obtained when Andrographis was planted at maximum density (40 plants m^-2^) (Fig 4B). Number of stalks was significantly different at initial stage of flowering, the highest number of stalks was recorded at 15 plants m^-2^ (Fig 4C). Notably, a similar trend to number of leaves was also found for plant width which reached the highest values at 15 and 30 plants m^-2^ during flowering stage (Fig 4D).

**Fig 5:**
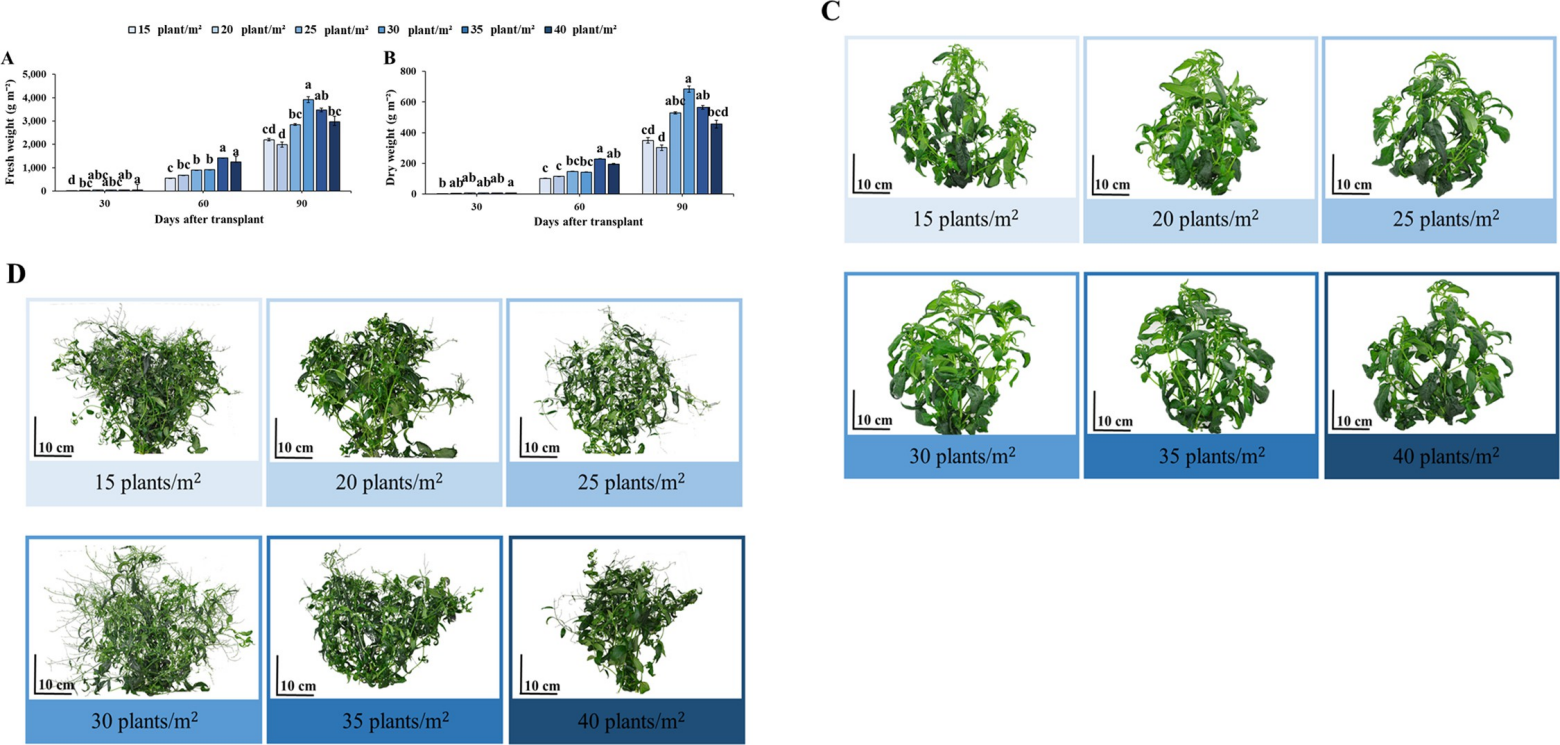
Effects of planting density on fresh weight (A) and dry weight (B) in g per one m^2^ of Andrographis during vegetative (30 DAT), initial flowering (60 DAT) and flowering (90 DAT) stages. The phenotypes of Andrographis grown at different planting densities at 60 (C) and 90 DAT (D). Values are represented as mean ± SE (*n* = 4). Different letters indicate significant difference between planting densities at p < 0.05. “ns” indicates no significant difference.

Increased planting densities were associated with increases in fresh and dry weight. At all planting densities, fresh weight yield per plant was significantly higher at 15 and 30 plants m^-2^ planting density (S2 Table). However, fresh weight yield per m^2^ was highest when Andrographis was cultivated at the moderate density of 30 plants m^-2^ (Fig 5A). The morphology of Andrographis plants at initial flowering stage and flowering stage is displayed in Fig 5C and 5D. Changes in dry wight yield at different planting densities showed similar trends as fresh weight yield (Fig 5B).

**Fig 6:**
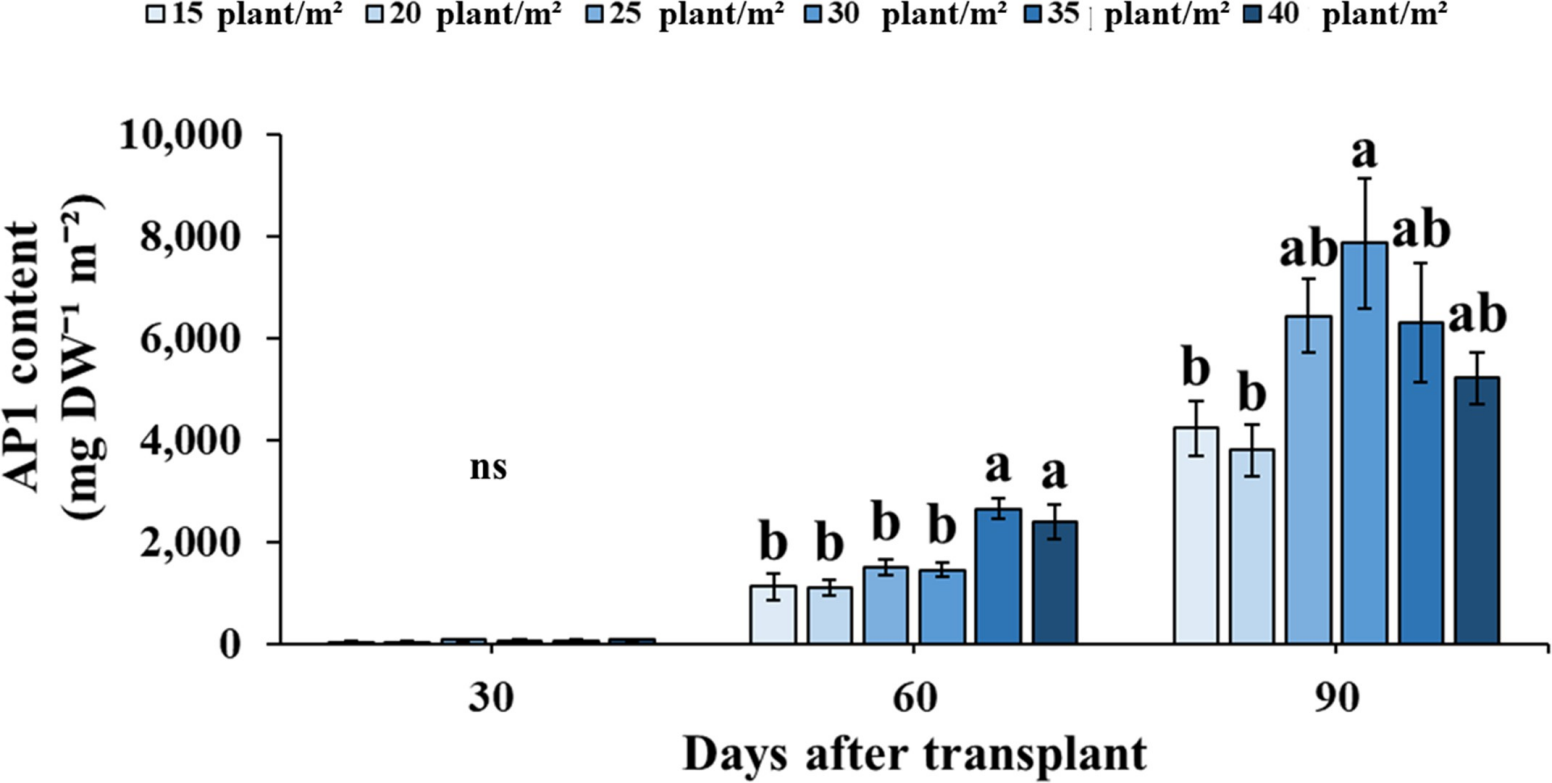
Andrographolide (AP1) content (mg DW^-1^ m^-2^) of Andrographis leaves at 6 planting densities during vegetative (30 DAT), initial flowering (60 DAT) and flowering (90 DAT) stages. Values are represented as mean ± SE (*n* = 4). Different letters indicate significant difference between planting densities at p < 0.05. “ns” indicates no significant difference.

AP1 content (expressed mg g^-1^ DW^-1^) among Andrographis plants was not affected by different planting densities across the three developmental stages (S2 Table). When AP1 content was expressed in mg DW^-1^ m^-2^, the accumulation of AP1 increased with increasing plant density for initial flowering stage and flowering stage (Fig 6). At initial flowering stage, the AP1 content was significantly higher at densities of 35 and 40 plants m^-2^ than at low densities of 15, 20, 25 and 30 plants m^-2^. Notably, there was a significantly higher accumulation of AP1 content in mg DW^-1^ m^-2^ at the plant density of 30 plant m^-2^ during flowering stage.

### Reflectance indices and principal component analysis

**Fig 7:**
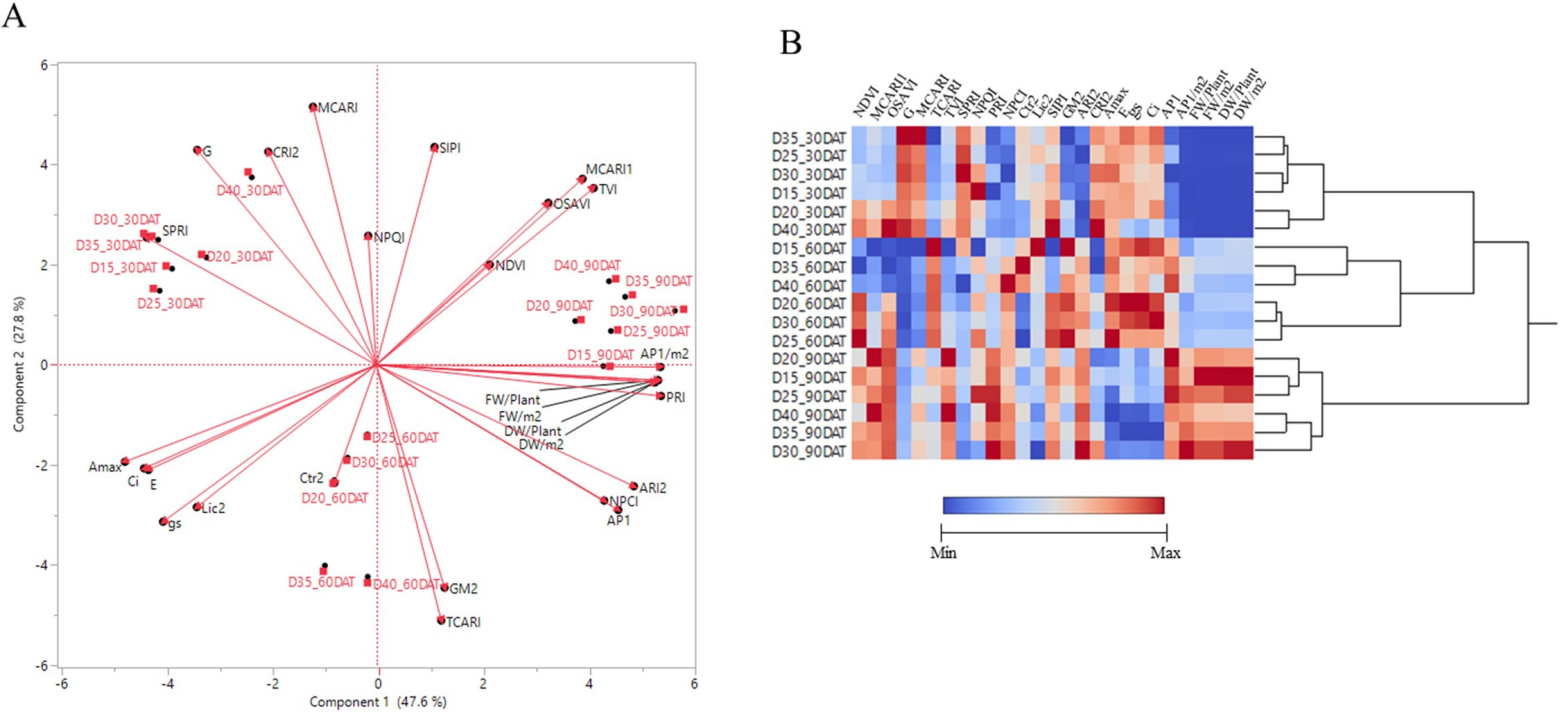
The relationship between 17 vegetation indices, eight physiological parameters and two types of AP1 measured among different density treatments during three developmental stages. Biplot of the PCA model of all samples (A). Heat mapping and clustering analysis of all measured variables using Z-scores for the normalized value (B). Color represents values of each parameter, with dark blue for low values and dark red indicating high values.

Correlational analysis and principal component analysis (PCA) were conducted to investigate relationships between 17 reflectance indices, gas exchange parameters, yield, and AP1 content during the three developmental stages (Fig 7A). A PCA biplot separates the six density treatments into three groups according to development stage. The contribution of first principal component (PC1) was 47.6% while PC2 was 27.8%. PC1 showed positive factor loadings for PRI index, AP1 per m^2^, FW per plant, FW per m^2^, DW per plant and DW per m^2^, while PC2 indicated positive factor loading for MCARI, NPQI and SIPI. Among these parameters, the correlations are shown in S3 Table. All photosynthetic traits and the 17 reflectance indices, *Pn* showed strong negative correlations with TVI (*r* = -0.85, *P* < 0.001), MCARI1 (*r* = -0.82, *P* < 0.001) and PRI1 (*r* = -0.81, *P* < 0.001). *gs* was also negatively correlated with MCARI1 and TVI (*r* = -0.84 and -0.86, respectively at *P* < 0.001). Moreover, AP1 content in mg g^-1^ DW^-1^ showed strong positive correlations with ARI2 (*r* = 0.89, *P* < 0.001), NPCI (*r* = 0.85, *P* < 0.001) and PRI (*r* = 0.82, *P* < 0.001), while the negative correlations were found with G (*r* = -0.86, *P* < 0.001) and SPRI (*r* = -0.84, *P* < 0.001).

Heat mapping and hierarchical clustering analyses of the 17 reflectance indices, gas exchange parameters, yield accumulation and AP1 content for planting densities during different developmental stages is shown in Fig 7B. These parameters are classified into six according to developmental stages (Fig 7B). The first and second clusters contained vegetative stage measures featuring the planting densities 15, 30, 35 and 40 plants m^-2^, and 20 and 40 plants m^-2^, respectively. The third and fourth clusters contained measures for initial stage of flowering. Notably, the fifth and sixth clusters were grouped into flowering stage, planting densities at 15, 20 and 25 plants m^-2^ were grouped in the fifth cluster, while the sixth cluster contained planting densities of 30, 35 and 40 plants m^-2^.

## Discussion

A key goal in commercialized plant production is to optimize growth and the biosynthesis of desirable secondary metabolites. However, environmental factors, and agricultural practices can strongly impact theses outcomes [53, 54]. Among these, planting density is the most effective factor influencing plant growth and crop quality [15, 55]. In the current study, we observed that plant density had a significant influence on the physiological responses, growth attributes, and plant biomass at different stages. Our study indicated that physiological parameters such as photosynthetic rate, internal CO_2_ concentration, transpiration rate, plant width and number of leaves were significantly affected by planting density (Figs 3 and 4). Leaf gas exchange parameters are the most important indices for evaluating potential photosynthetic productivity [56]. Photosynthesis in plants directly affects plant growth, development and crop yield, which are influenced by both stomatal and non-stomatal factors [57]. Our findings showed that increases in planting density at 20 to 40 plants per m^2^ result in decreased photosynthetic rate (*Pn*) of Andrographis leaves during flowering stage (90 DAT). This result was consistent with previous reports showing dramatic decreases in photosynthetically active radiation at high plant densities [55, 58].

According to our findings of plant density on growth responses and yield accumulation of Andrographis grown in hydroponic PFAL systems, lowest (15 plants m^-2^) and mid planting densities (30 plants m^-2^) during flowering stage (90 DAT) affected growth responses by increasing plant width and number of leaves, while growth responses declined when density increased to 35 and 40 plants m^-2^ (Fig 4B and 4C). Our results corroborate previous reports [59, 60] showing that leaf area index (LAI) and leaf number increases with plant density. In the present study, we found that the increase in LAI and leaf number, especially at higher planting densities, decreased the light intensity inside the plant canopy at 90 DAT (S2 Fig). Such effects have been shown to result in a reduction in light intensity and changes in plant organ morphology [60, 61]. Moreover, studies in various plant species have shown that increases in plant density may result in changes in organ development and leaf morphology such as lamina width internode diameter and sheath extension [14], alongside changes in biomass accumulation in rapeseed (*Brassica napus* L.) [15] and *Eucalyptus globulus* [62]. High planting densities at maturing stages may create a dead air zone with low air movement, subsequently increasing boundary layer resistance and lowering CO_2_ diffusing rates into the inner and outer canopies [63]. Increases in CO_2_ concentration may therefore help to distribute CO_2_ gas inside plant canopies, and subsequently enhance net CO_2_ assimilation rates. Interestingly, different plant densities did not affect plant height and number of stalks during developmental stage of Andrographis. However, the relationship between plant density, plant height and number of stalks grown under PFAL systems still needs to be further researched.

In our study, plant density affected plant biomass in Andrographis plants under a hydroponic PFAL system. The FW and DW yield per plant grown at 15 and 30 plants m^-2^ plant density provided the highest plant biomass (S2 Table). The trend of whole plant FW and DW yield at flowering stage mirrored the data for number of leaves (Fig 4D), corroborating other reports [60, 64] in *A*. *paniculata*. Moreover, when expressed per m^2^ of FW and DW, the results showed that a planting density of35 plants per m^2^ was associated with the highest productivity of FW and DW yield per m^2^ during flowering stage (Fig 5A and 5B). The result indicates that there was a reduction of FW and DW per m^2^ at high planting densities (35 and 40 per m^-2^) during flowering stage which may be explained by intense intraspecific competition for resources (water, nutrient, flow rate and light).

Environmental factors, and agricultural practices are important for enhancing the quality of secondary metabolites [65]. Andrographolide (AP1), the major secondary metabolite in Andrographis, has numerous pharmacological effects [66–68]. Findings in Liu [54] and Verma [60] found that the synthesis of secondary metabolites in *Panax notoginseng* and *Andrographis paniculata* were enhanced when growing at high densities. Although the accumulation of AP1 content in mg g^-1^ DW^-1^ is not affected by planting density across developmental stages (S2 Table), when AP1 content was expressed as mg DW^-1^ m^-2^, the production of AP1 content was significantly higher at 30 plants per m^2^ compared with other planting densities (Fig 6).

A vegetation index is a widely used method for evaluating physiological responses and crop state estimation by calculating a single metric from multiple spectral bands [69]. Moreover, the vegetation indices proved useful for several predictable traits [70]. In the present study, metrics of the 17 reflectance indices, gas exchange parameters, yield, and AP1 content among the six planting densities, were clearly divided into three major clusters: vegetative stage (30 DAT), initial stage of flowering (60 DAT) and flowering stage (90 DAT). However, planting density at flowering stage was divided into two subgroups (30, 35 and 40 plants m^-2^ and 15, 20 and 25 plants m^-2^) (Fig 7B). This is consistent with Chutimanukul *et al* [33] who examined the influence of light spectra on holy basil plants at different developmental stages. This result suggested that development stage appears to be the key determinant of physiological responses and vegetation indices. In the present study, stages of plant growth and development has been shown to affect the vegetation indices (Fig 7B). Interestingly, AP1 accumulation and vegetation indices were related to plant developmental stage, suggesting direct correlation between AP1 content and growth stage. Correlation analysis further suggested that vegetation indices analysis for ARI2 and G are the first two highest correlation with AP1 content at r = 0.89 and r = -0.86 at P < 0.001, respectively. ARI2 is a reflectance measurement that is sensitive to anthocyanin in plant while leaf greenness is measured by G index. ARI2 is related to the phenolic pigment, which is the functional secondary metabolites, like AP1 is one of the secondary metabolite groups. However, chlorophyll is often referred to leaf greenness, which is the primary pigment used in photosynthesis. Several studies have shown that chlorophyll content was significantly correlated with secondary metabolites such as total phenolic compounds, flavonoid content and anthocyanin content. Our results showed a strong positive correlation between ARI2 and AP1 content, and negative correlation between G and AP1 content. As the result demonstrates, ARI2 and G had a high potential index to determine the AP1 content which showed the wavelength between 500 to 800 nm. Consequently, ARI2 and G indexes might be used to estimate AP1 accumulation in Andrographis leaves. In addition, our result showed that PRI showed a strong positive correlation with plant fresh weight (r = 0.93, P < 0.001). Thus, PRI index may be able to predict the harvesting stage of Andrographis plant grown under controlled environment. These results suggest that basic assessment of leaf reflectance can be used as a non-destructive estimation of plant secondary metabolites in Andrographis. Moreover, these findings provide valuable information to optimize the growth and AP1 content of Andrographis grown in hydroponic PFAL and should be of value for the pharmaceutical industry.

## Conclusion

This study investigated the effects of plant density on physiological responses, yield and AP1 content in Andrographis plants under hydroponic cultivation in a controlled environment. The present study can be seen as a first step toward developing agrotechniques for industrialized Andrographis production under controlled conditions in PFAL system to achieve optimal yields and quality. Results indicate that a planting density of 30 plants m^-2^ had a beneficial influence on plant growth, yield and AP1 content per unit area. Furthermore, correlation analysis of vegetation indices indicated ARI2 and G were positively correlated with AP1 content and it can support the advantages of using a non-destructive approach to predict plant responses and biochemical traits in Andrographis. This should be of great value in Andrographis plant production in PFAL technology, and provide useful information for pharmaceutical industry.

## Supporting information

S1 Fig The conceptual framework of the study to investigate the physiological responses, yield and AP1 content of Andrographis in response to plant density. (DOCX)

S2 Fig The light intensity level of Andrographis at six planting densities during vegetative (30 DAT), initial flowering (60 DAT) and flowering (90 DAT) stages. Values are represented as mean ± SE (*n* = 4). “ns” indicates no significant difference.
(DOCX)

S1 Table The details of the daily environment under PFAL. (XLSX)

S2 Table Effect of planting densities on productivity and andrographolide (AP1) content (mg g^-1^ DW^-1^) of Andrographis during vegetative (30 DAT), initial flowering (60 DAT) and flowering (90 DAT) stage. (DOCX)

S3 Table The correlation analysis among leaf reflectance, gas exchange parameters, yield and andrographolide content (AP1) of *Andrographis paniculata* (Burm. F.) under six planting densities during three developmental stages. (DOCX)
