# Supplementary material for: Physiological responses, yield and medicinal substance (andrographolide, AP1) accumulation of Andrographis paniculata (Burm. f) in response to plant density under controlled environmental conditions
Source: PLoS One. 2022 Aug 4;17(8):e0272520. doi: 10.1371/journal.pone.0272520 (PMC9352076; doi:10.1371/journal.pone.0272520)
Supplement: S1 Fig — (DOCX) [file pone.0272520.s001.docx]

**Supplementary Figure 1:**

**
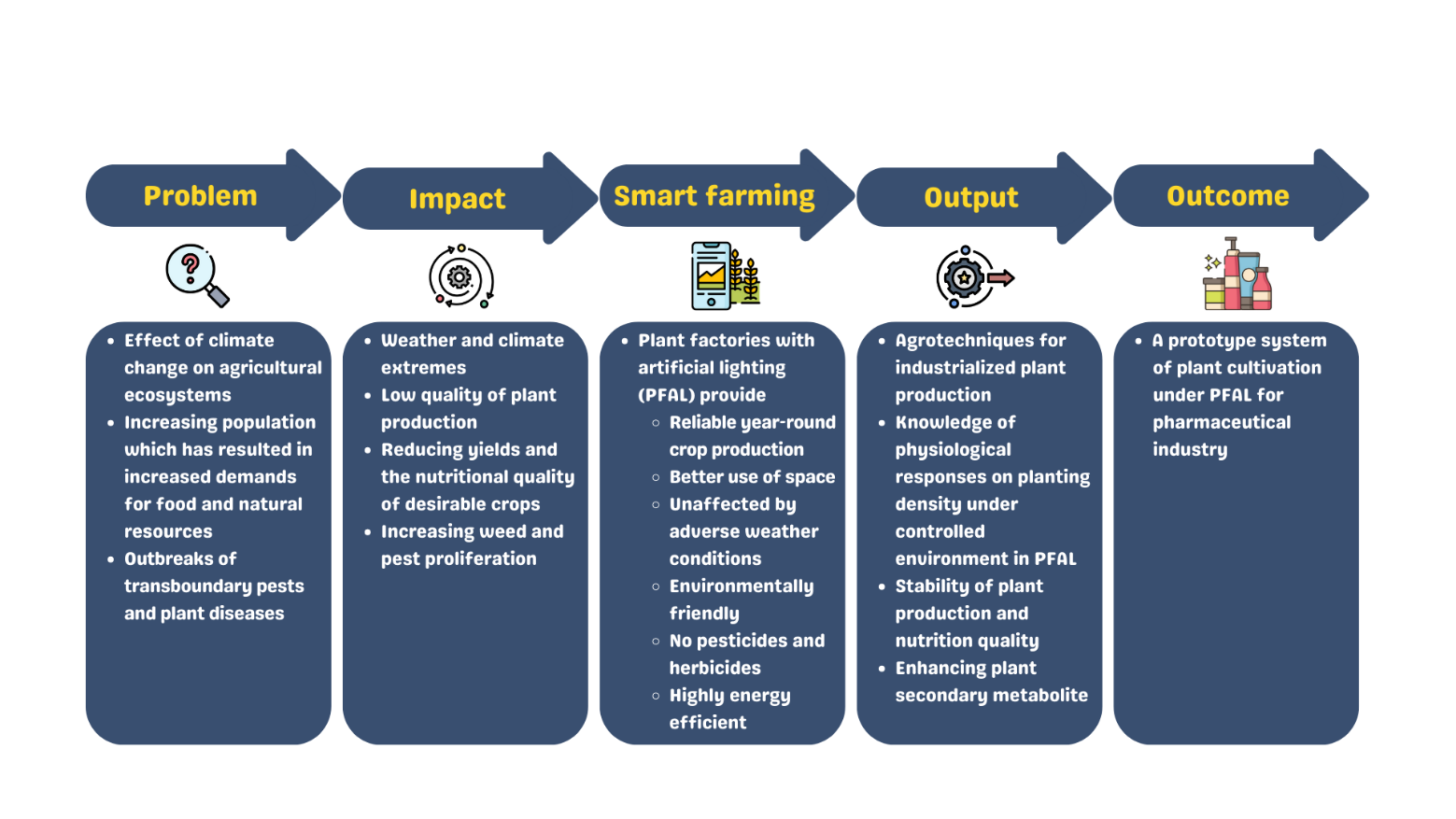
**

**S1 Figure.** **The conceptual framework of the study to investigate the physiological responses, yield and AP1 content of Andrographis in response to plant density**.
