# Supplementary material for: Physiological responses, yield and medicinal substance (andrographolide, AP1) accumulation of Andrographis paniculata (Burm. f) in response to plant density under controlled environmental conditions
Source: PLoS One. 2022 Aug 4;17(8):e0272520. doi: 10.1371/journal.pone.0272520 (PMC9352076; doi:10.1371/journal.pone.0272520)
Supplement: S2 Fig — Values are represented as mean ± SE (n = 4). “ns” indicates no significant difference. (DOCX) [file pone.0272520.s002.docx]

**Supplementary Figure 2:**


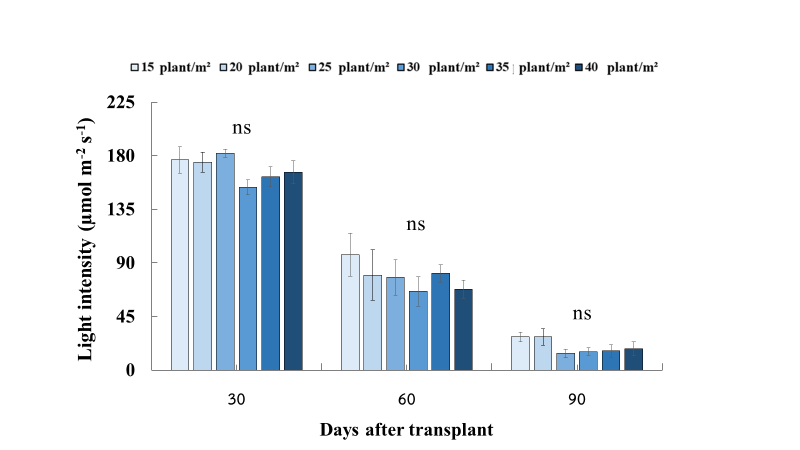


**S2 Figure.** **The light intensity level of Andrographis at six planting densities during vegetative (30 DAT), initial flowering (60 DAT) and flowering (90 DAT) stage. Values are represented as mean ± SE (*n* = 4). “ns” indicates no significant difference.**
