# Supplementary material for: Physiological responses, yield and medicinal substance (andrographolide, AP1) accumulation of Andrographis paniculata (Burm. f) in response to plant density under controlled environmental conditions
Source: PLoS One. 2022 Aug 4;17(8):e0272520. doi: 10.1371/journal.pone.0272520 (PMC9352076; doi:10.1371/journal.pone.0272520)
Supplement: S2 Table — (DOCX) [file pone.0272520.s004.docx]

**Supplementary Table 2:**

**Effect of planting densities on productivity and andrographolide (AP1) content (mg g^-1^ DW^-1^) of Andrographis during vegetative (30 DAT), initial flowering (60 DAT) and flowering (90 DAT) stage.**

| Day after  transplanting | Density  (plant m^-^²) | Fresh weight  (g plant^-1^) | Dry weight  (g plant^-1^) | AP1 content  (mg g^-1^ DW^-1^) |
| --- | --- | --- | --- | --- |
| 30 DAT | 15 | 1.43±0.39 | 0.27±0.08 | 6.99±0.64 |
|  | 20 | 1.21±0.14 | 0.25±0.04 | 6.95±0.93 |
|  | 25 | 1.57±0.09 | 0.36±0.05 | 8.41±0.57 |
|  | 30 | 1.38±0.05 | 0.28±0.02 | 6.06±1.36 |
|  | 35 | 1.33±0.28 | 0.26±0.05 | 6.54±0.37 |
|  | 40 | 1.30±0.32 | 0.23±0.07 | 7.76±0.42 |
|  |  | ns | ns | ns |
| 60 DAT | 15 | 36.31±3.88 | 6.83±1.22 | 10.71±0.53 |
|  | 20 | 34.12±5.75 | 5.82±0.91 | 9.78±1.39 |
|  | 25 | 35.79±1.58 | 5.94±0.24 | 10.16±0.79 |
|  | 30 | 30.44±4.23 | 4.77±0.65 | 10.27±0.50 |
|  | 35 | 40.37±2.25 | 6.57±0.33 | 11.53±0.46 |
|  | 40 | 31.06±4.00 | 4.91±0.61 | 12.13±0.38 |
|  |  | ns | ns | ns |
| 90 DAT | 15 | 146.60±12.69 a | 23.32±2.30 a | 12.04±0.43 |
|  | 20 | 99.46±9.23b c | 15.13±1.77 bc | 12.55±0.77 |
|  | 25 | 113.71±5.81 b | 21.16±3.34 b | 12.45±0.77 |
|  | 30 | 130.42±13.95 ab | 22.80±2.78 ab | 11.43±0.90 |
|  | 35 | 99.25±8.37 bc | 16.17±1.56 bc | 10.91±0.94 |
|  | 40 | 74.06±5.87 c | 11.41±1.05 c | 11.45±0.42 |
|  |  | * | * | ns |
