# Supplementary material for: Physiological responses, yield and medicinal substance (andrographolide, AP1) accumulation of Andrographis paniculata (Burm. f) in response to plant density under controlled environmental conditions
Source: PLoS One. 2022 Aug 4;17(8):e0272520. doi: 10.1371/journal.pone.0272520 (PMC9352076; doi:10.1371/journal.pone.0272520)
Supplement: S3 Table — (DOCX) [file pone.0272520.s005.docx]

**Supplementary Table 3:**

**Th****e correlation analysis among leaf reflectance, gas exchange parameters, yield and andrographolide content (AP1) of *Andrographis paniculata* (Burm. F.) under six planting densities during three developmental stages.**

**
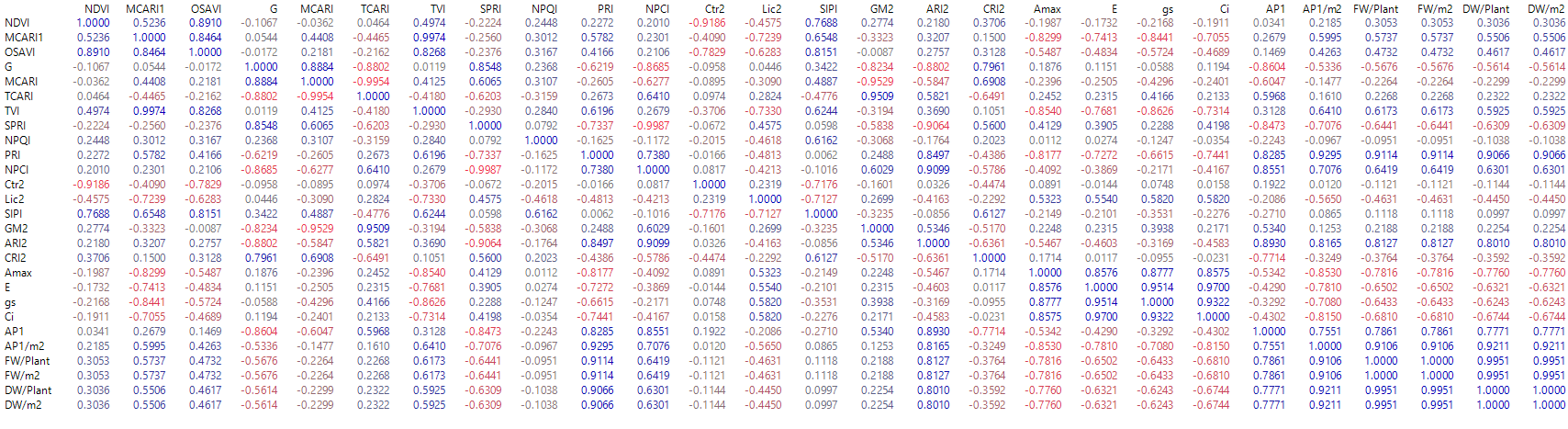
**
